# Buffalo Medical Association

**Published:** 1880-04

**Authors:** 


### Society Reports.

---

### BUFFALO MEDICAL ASSOCIATION.

*Stated Meeting, March 2, 1880.*

THE PRESIDENT, DR. LUCIEN HOWE, IN THE CHAIR.

THE subject for special consideration was presented in a paper by Prof. J. F. Miner, entitled "The Diagnosis of Disease without the aid of Physical Signs."

He pointed out the exact conclusions at which formerly physicians arrived, by depending upon the general symptoms of their patients, and hinted that perhaps the pendulum of medical opinion was now swinging too far, in favor of "Physical Signs."

He would not undervalue the assistance to be derived from the stethoscope or other instruments of precision, but thought that by depending too much upon the evidence thus furnished we were in danger of neglecting many important symptoms upon which a diagnosis could be readily and surely based.

On motion of Dr. Rochester, the discussion of this subject was deferred till another meeting, there being another of much importance to the profession of this locality, which demanded immediate consideration.

Dr. White then introduced the following preambles and resolutions:

Whereas, It is the duty of the medical men in every community in which they reside, to warn all those who rely upon them for any medical advice of any danger, present or threatened, to the public health of which they are cognizant; and

Whereas, We know there exists in the very centre of this city an immense open cess-pool—a large accumulation of decaying animal and vegetable matter—known as the Hamburg Canal and its connections; and

Whereas, This sink of filth has long existed, and notwithstanding all efforts thus far made at palliation, is steadily increasing in extent, and in the amount of poisonous effluvia emitted therefrom; and

Whereas, The emanations from this vile collection, in the opinion of the members of this Society, now exert a highly deleterious influence upon all the inhabitants living in its neighborhood; and

Whereas, In case of the prevalence of any epidemic disease, the health of the whole city would be greatly endangered by this mass of uncovered fermenting material, and also from the unsanitary condition of the sewerage in the lower part of the town; and

Whereas, Much time is necessarily required to adopt and execute any comprehensive plan for the permanent drainage of the city: therefore,

Resolved, That this Society urge the Common Council, the State authorities having charge of the canal, the Board of Health of the city, and all good citizens immediately to take measures not only to remedy the terrible nuisance of the Hamburg Canal, but to adopt a comprehensive plan of drainage, by means of which the entire city, from North street southward, all of which inclines in the same direction, and should be embraced in the same plan, may be effectually and permanently drained.

Resolved, that we earnestly entreat the responsible authorities, and especially the Common Council, not longer to neglect this great sanitary work, in the delusive hope that through the influence of the abundance of pure air always blowing over the city, we shall much longer escape the legitimate effects of the pestiferous emanations from this sink of corruption upon the health of our inhabitants.

Resolved, That in the opinion of the members of this Society the palliative measures heretofore resorted to, instead of correcting the evil do but increase it by spreading it over a larger surface, polluting at the same time the water flowing in the canal for many miles, rendering it utterly unfit for use by "man or beast," and exposing to its horrible exhalations all who navigate this important channel or reside in its vicinity.

Resolved, That in the opinion of this Society no remedy can afford permanent relief from the baleful effects of the want of proper sewerage of this extensive territory, which does not contemplate its removal into the river below the water supply for the city, keeping it entirely out of the harbor and the canal.

Resolved, That as the guardians of the public health, as sentinels on sanitary duty, we admonish our fellow citizens that the apathy, the supineness which prevails in reference to this dreadful pest-hole must be shaken off, or the day is not distant when, like poor neglectful Memphis, we shall repent our omission in dust and ashes.

Resolved, That a copy of this preamble and the accompanying resolutions be transmitted to the Common Council, the Board of Health, and to the secular papers, with the request that they be published therein.

In moving the adoption of these resolutions, Dr. White said that this Society can perhaps do no more than point out the evil, the responsibility of remedying it lies with the Common Council, the Board of Health, the City Engineer, and the thinking business men. He would, however, suggest that instead of endeavoring to work out this problem of sewerage without the assistance of experts, those who have studied the subject should be called on for advice. Sanitary engineering, though comparatively a new study, is now recognized as a specialty.

Memphis had been so bitterly taught the necessity of sanitary precaution that she has now wisely employed Col. Waring, who has already proposed a plan which, it is believed, will remedy her heretofore sadly defective system of drainage.

There was a rumor that the Central Railroad had offered to fill up the ditch and construct a good sewer the whole length of it, but this would not rectify the trouble. Clearly, some other measures must be resorted to for perfecting a system of drainage, and without doubt the enlightened part of the community were ready for action.

Dr. George N. Burwell seconded the motion to adopt the resolutions, stating their sentiment fully expressed his own views. The Hamburg Canal had become perverted from its original purpose, and was now an unmitigated evil. It could readily give rise to a great epidemic, should there be at any time a high temperature accompanied by scarcity of rain. The seeds of disease were generated by a hot, dry season. Memphis bought the knowledge of this principle at a dear cost, and it behooves the people of Buffalo to evince their wisdom by taking prompt action.

Dr. Frederick W. Bartlett had proved from actual experience that in the district adjacent to the Hamburg Canal, there was an excess of mortality in cases of diphtheria, typhoid and scarlet fevers, which could only be attributable to the vicinity of such a nuisance.

Dr. Thomas F. Rochester said the canal was in effect nothing but a receptacle for disease-producing elements. In England scientific investigation had demonstrated that the drainage from sewers can be disinfected and diverted into any channel without detriment to the public health. This method might be adopted in the case now under consideration, should the entire removal of the nuisance be found impracticable.

Dr. S. S. Greene endorsed the resolutions, being convinced that the noxious gases arising at the head of the canal were swept by westerly winds over the city, distributing disease.

An invitation being formally extended to those gentlemen present who were not members of the Association to join in the discussion, Mr. Young, the City Engineer, said he believed the only practicable system of drainage, affording permanent relief, would be a belt sewer, connecting with a tunnel under the Erie Canal to the Niagara River. The enormous expense, however, of this plan might make it objectionable. He had inspected the ground and judged that a survey could be made within two weeks, and the results submitted. In his opinion the swift current of the Niagara River would effectually prevent the pollution of the water by the sewer discharge.

Mr. E. S. Hawley was gratified that the attention of medical men had been directed to this matter, as the public would respect their opinions, and in view of the present city debt, strenuous efforts would be required to accomplish the project. An intermediate course would only give temporary relief.

Mr. P. P. Pratt concurred in all the foregoing remarks, especially supporting the measure for the employment of competent engineers as a primary step in the work.

Dr. A. H. Briggs, the Health Physician, had been surprised at the comparative health of the city during his ten years' practice. He was satisfied the Hamburg Canal had caused and rendered extraordinarily fatal, such diseases as typhoid fever, diphtheria, &c.

Dr. A. R. Davidson thought that even if the Niagara River became polluted by the canal sewerage directed into it, this would again be thoroughly oxydized by air and water before reaching Lake Ontario.

Dr. E. C. W. O'Brien believed the evil effects of the canal had not been over-estimated. Much opposition would be raised on account of the expense. The City Engineer proposing such a plan must be hopeless of re-election.

Dr. J. F. Miner heartily approved the resolutions, it being undeniable that an enlightened community had never endured a more intolerable nuisance than the Hamburg Canal.

Dr. J. C. Cronyn thought the canal had unquestionably produced great mortality in its vicinity. The present current-producing plan was such an absurd and abominable piece of engineering that the originators ought to refund its whole expense.

After this discussion the resolutions were unanimously adopted, and the meeting adjourned.

